# Healthcare capacity strengthening in conflict settings through virtual emergency medical training and outreach: Ukraine and Sudan case studies

**DOI:** 10.3389/fpubh.2024.1441322

**Published:** 2024-10-18

**Authors:** Shawn M. D’Andrea, Nada Fadul, MarkAlain Dery, William L. Brim, Andrea M. Israel, Bruce Baird Struminger

**Affiliations:** ^1^Department of Emergency Medicine, University of Botswana, Gaborone, Botswana; ^2^Department of Internal Medicine, Sustainable Development Response Organization, University of Nebraska Medical Center College of Medicine, Omaha, NE, United States; ^3^Swiss Foundation for Innovation, New Orleans, LA, United States; ^4^Center for Deployment Psychology, Uniformed Services University of the Health Sciences, Bethesda, MD, United States; ^5^ECHO Institute and Department of Internal Medicine, University of New Mexico Health Sciences Center, Albuquerque, NM, United States

**Keywords:** health emergencies, digital learning, war, humanitarian response, capacity strengthening, conflict

## Abstract

The use of digital learning in healthcare is expanding in a range of contexts including for settings of armed conflict. Humanitarian emergencies and war often lead to a surge of traumatic injuries, emotional distress, and disruption to health systems risking neglect and exacerbations of chronic illness, and acute infectious disease outbreaks, often requiring an international response. On the ground humanitarian response is often essential though logistical and security challenges can delay these responses, and the reliance on an international workforce unfamiliar with local cultures can create challenges in response efforts. In crises where local healthcare workers have limited training, or experience in emergency care, digital health care education can augment in-person response and training efforts. In recent years digital emergency care education programs have been deployed to both Ukraine and Sudan. A review of each of these programs demonstrates successes in and potential utility of remote healthcare capacity strengthening through digital education in settings of war. These programs provide important lessons in strengths of and challenges in developing and delivering just in time learning programs to settings of active armed conflict suggesting similar potential utility in a variety of humanitarian emergency contexts.

## 1 Introduction

In May 2023, the 76th World Health Assembly (WHA) adopted Resolution 76.2; “Integrated Emergency, Critical, and Operative Care for Universal Health Coverage and Protection from Health Emergencies.” This resolution calls on WHO member states to bolster their capabilities in these areas noting that “emergency, critical and operative care embedded within the broader health system is vital to maintaining the continuity of essential health services in fragile and conflict-affected settings” (1). To meet the resolution’s aims, it is crucial to develop innovative strategies that complement traditional humanitarian responses and enhance health system strengthening. We highlight two emergency response projects that used virtual emergency care training as a timely adjunct to on-ground efforts, improving emergency care in conflict settings. These programs are characterized by rapid deployment, cost-efficiency, reduced security risks, and the utilization of a global pool of content experts including diaspora of the affected countries.

Humanitarian emergencies often lead to a surge of traumatic injuries (2), emotional distress (3), disruption to health systems risking neglect and exacerbations of chronic illness (4), and acute infectious disease outbreaks (5), requiring an international response. These emergencies are complex, involving multiple sectors and international responders, and necessitate a multifaceted approach.

The role of international humanitarian response is essential to save lives and reduce suffering during complex emergencies. Humanitarian response organizations provide critical services and materials including vital, lifesaving resources and personnel. However, logistical and security challenges can delay these responses, and the reliance on an international workforce unfamiliar with local cultures can create challenges in response efforts. Despite their irreplaceable and essential nature, these missions are expensive and financially limited. With these challenges in mind, there is a significant opportunity to augment on-ground efforts with digital and remote capacity strengthening, which can mobilize certain global human resources more swiftly and effectively than traditional methods.

In most humanitarian emergencies, local healthcare workers (HCWs) are indispensable, providing continuous care throughout all phases of a crisis (6). Often, these workers must deliver emergency medical and psychological trauma care without prior training. While natural disasters such as earthquakes and floods cause immediate mortality and morbidity, highlighting the need for rapid emergency medical response, protracted crises, especially when complicated by disruptions to the healthcare system, can lead to prolonged trauma and medical needs. Despite the presence of international agencies, local HCWs are critical in delivering consistent care and often require sustained training, consultation, and support in emergency medical and psychological interventions. Furthermore, as emergency care requires a multidisciplinary response of health professionals, HCWs from a range of backgrounds and specialties may benefit from emergency care training. In such circumstances, providing just-in-time emergency care training for local HCWs is a priority.

## 2 Ukraine armed conflict trauma training (ACCT)

In June 2022, shortly after the onset of the ongoing Ukraine-Russia conflict, the Swiss Foundation for Innovation (SFI) in collaboration with the Ukraine Ministry of Health (MOH) identified a pressing need for trauma care training for healthcare workers (HCWs) in Ukraine, particularly those in prehospital roles. The SFI team initiated a partnership with Project ECHO at the University of New Mexico, and together they rapidly developed an emergency trauma care distance learning course concept. This was followed by a series of rapid information-gathering meetings with leaders from various sectors including humanitarian, disaster medicine, and global health. Notable participants included the World Health Organization (WHO), the American College of Emergency Physicians, the Harvard Humanitarian Initiative, and the Uniformed Services University Center for Deployment Psychology. From these meetings and a review of existing trauma care education curricula, the Project ECHO-SFI teams crafted a course curriculum based on the WHO-International Committee of the Red Cross (ICRC) Basic Emergency Care (BEC) trauma module; additionally, this program integrated a 10-min segment into each learning session focused on trauma mental health. This comprehensive program, named the Ukraine Armed Conflict Trauma Training (ACTT), was launched within just 15 days from the initial planning session.

### 2.1 Ukraine ACTT course overview

The Ukraine ACTT course comprised 16 one-hour training sessions conducted over 8 weeks, starting in June 2022 and ending in August 2022. Each session began with a 20–30 min didactic presentation based on the ICRC/WHO BEC Trauma module curriculum. Instructors were asked to follow the BEC Trauma module content and sequence though were also welcomed to share their own experience and elaborate on topics based on their area of expertise. This was followed by a discussion between the program moderators and the didactic presenter informed by questions from the participants submitted through the webinar Q&A feature. Each session concluded with a 10-min trauma mental health training segment delivered by specialists from the Uniformed Services University’s Center for Deployment Psychology. The course utilized the Zoom webinar platform, as opposed to the Zoom meeting format, to address potential security concerns that could arise from participant visibility in a meeting format. The webinar allowed for visual instruction and interaction between presenters and moderators while limiting participant communication to a text-based question and answer format. Simultaneous Ukrainian-English interpretation was provided, enhancing accessibility. A core team of four SFI personnel collaborated closely with the Ukraine MOH to recruit participants, monitor the course’s impact, and gather feedback. The design of this just-in-time virtual learning program was informed by the 20 years of experience of the UNM Project ECHO team in designing and delivering remote continuing professional development programs, including many learning programs related to the global COVID-19 pandemic response (7–11).

### 2.2 Ukraine ACTT instructor recruitment

A significant advantage of the remote training program was the immediate availability and accessibility of a global pool of WHO/ICRC BEC certified instructors ready to assist. Recruitment efforts were initially directed through email invitations to this roster and other specialists from the networks of planning team members, prioritizing Ukrainian-speaking experts. Subsequently, the recruitment widened to include trauma and emergency care specialists across various disciplines, aiming to engage skilled medical educators. This strategy successfully assembled a diverse faculty of instructors from Ukraine, Tanzania, India, and the United States, each bringing valuable perspectives to the educational sessions.

### 2.3 Ukraine ACTT trauma mental health component

The concept of including a trauma mental health component in the training program was a strategic decision made during the program planning phase. Despite the short timeline available, a team from the Center for Deployment Psychology (CDP) at the Uniformed Services University in the United States committed to providing a longitudinal, 16-part trauma mental health program with the time allotted of 10 min per session and one session with the full 1-h program dedicated to mental health trauma education. This team provided didactic education and shared resources for HCWs working in settings of armed conflict to address mental health trauma principles for patients as well as the mental health and well-being of the HCWs. Given the challenge of limited didactic time, the team took a microlearning approach to the curriculum (12) and developed small units of training focused on acquisition of knowledge and skills. Topics were designed to be utilized by prehospital HCWs without prior in-depth mental health training. The goal was to provide information and resources sufficient for immediate implementation by HCWs in the field. Quick response (QR) codes were embedded with links to web-based resources that could be used in the field. Whenever possible, the CDP team intentionally linked mental health content with the focus of the trauma topic subject for the overall session. For example, mental health aspects of amputation were provided following the extremity BEC didactic and assessment of Traumatic Brain Injury (TBI) using the Military Acute Concussion Examination followed the blast didactic (Table 1).

**Table 1:** ACTT course curriculum with attendance.

Ukraine ACTT course syllabus with attendance				
Session	Trauma topic (BEC)	Mental health topic	Session date	Attendees
1	Primary survey ABCDE	Psych first aid (Part 1)	6/9/22	113
2	Trauma secondary/Sample Hx	Psych first aid (Part 2)	6/14/22	51
3	Breathing and airway management	Psych first aid (Part 3)	6/16/22	63
4	Head and neck	Psych first aid (Part 4)	6/21/22	61
5	Chest	Provider resilience (Part 1)	6/23/22	54
6	Abdominal	Provider resilience (Part 2)	6/28/22	55
7	Spinal cord, internal and pelvic	iCOVER	6/30/22	56
8	Extremity	Amputation	7/5/22	46
9	Crush	Sleep (CBTi – Combat Sleep)	7/7/22	50
10	Blast	TBI (MACE2)	7/12/22	53
11	Burn	Stress first aid (Part 1)	7/14/22	46
12	Triage	Stress first aid (Part 2)	7/19/22	37
13	Pediatric trauma	Age related reactions to trauma	7/21/22	38
14	Trauma in pregnancy	Combat injured families	7/26/22	11
15	CBRNE	Mortuary affairs	7/28/22	12
16	Clinical mental health	PTSD, ASD, MDD, AUD, suicide	8/2/22	12

Represents the course syllabus and title of each session emergency care and mental health content along with participant attendance per session.

### 2.4 Ukraine ACCT observations

The Ukraine ACTT program was pioneering as it represents one of the first rapidly developed and deployed remote trauma care training programs for HCWs in an active conflict setting. The program was delivered in 16 sessions offered twice weekly between June 9th and August 2nd 2023. This just-in-time capacity building program engaged 238 unique participants with a total of 758 attendances suggesting that there were repeated attendances among individual attendees. Among the unique participants (*N* = 238), 42% were nurses, nurse practitioners and physician assistants; 32.15% were first responders; 13.09% were medical doctors; and 3.83% were mental health providers (see Table 2 for details).

**Table 2:** Ukraine ACTT representation of professions among attendees.

Attendee profession	Percentage of attendees present
Nurse/nurse practitioner/physician assistant	42.53%
First responder (such as EMT, paramedic)	32.15%
Medical doctor	13.09%
Mental health provider (such as counselor, psychologist, social worker)	5.83%
Testing or laboratory personnel	2.42%
Community health worker	1.56%
Teacher/educator	1.28%
Public health official	0.43%
Other	0.28%
Other medical provider (such as pharmacist, dentist, veterinarian)	0.28%
Administrator	0.14%

Describes the professions represented among attendees for all sessions of the Ukraine ACTT program (*N* = 758).

Anonymous feedback was collected from the attendees after each of the learning sessions. With respect to knowledge acquisition, participants self-rated their knowledge acquisition on aggregate as significant: 5.24% of attendees who responded to the post-session surveys (*N* = 708) rated their knowledge as Extremely Knowledgeable before and this increased to 11.3% after; 24.93% of attendees rated their knowledge as Very Knowledgeable before and this increased to 52.60% after (see Table 3 for additional information). Attendees who completed the post-session surveys (N = 708) rated the relevance of the ACTT training program as overall highly relevant to their practice; data aggregated across all 16 learning sessions rated them as: Extremely Relevant (27.7%); Very Relevant (50.52%); Moderately Relevant (14.98%); Slightly Relevant (6.45%); and Not at All Relevant (0.35%) (Table 4).

**Table 3:** Ukraine ACTT rating of subject knowledge before and after the program by attendees.

Knowledge before Session	Percentage	Knowledge after session	Percentage
Extremely knowledgeable	5.41%	Extremely knowledgeable	11.30%
Very knowledgeable	24.93%	Very knowledgeable	52.60%
Moderately knowledgeable	52.14%	Moderately knowledgeable	31.90%
Slightly knowledgeable	16.52%	Slightly knowledgeable	4.30%
Not at all knowledgeable	1%	Not at all knowledgeable	0%

Summarizes post-session responses of attendees regarding level of knowledge of program content before and after sessions. This table represents aggregated data (*N* = 708) from all 16 sessions.

**Table 4:** Ukraine ACTT attendee rating of relevance to practice.

How relevant is the session to your current work?	Percentage
Extremely relevant	27.70%
Very relevant	50.52%
Moderately relevant	14.98%
Slightly relevant	6.45%
Not at all relevant	0.35%

Reflects participant responses when asked how relevant the session was to their current work (*N* = 708).

The virtual format’s rapid development and deployment represented a significant benefit, contrasting with the lengthy timelines typically associated with coordinating in-person training initiatives. The streamlined logistical process, including assessments of wireless connectivity and participant recruitment, was efficiently managed by a small team on the ground in Ukraine which also reduced personnel exposed to security risks. The choice of webinar format over a more interactive video conference platform was assumed to be warranted to mitigate security risks, though it may have limited participant interaction. Convenience and safety of participants in the program was an additional strength. Participants were able to join from a location of their choice and learning sessions were able to be viewed both live and on an asynchronous basis as all the learning sessions were recorded and made available on a private YouTube channel (https://www.youtube.com/playlist?list=PLM3v2ae2FB_xGA9dOP9LQ9vIsQ0e_xCQv) which was shared with participants. The involvement of the Ukraine MOH was found to significantly boost attendance and engagement, suggesting substantial added value of ministry involvement. Finally, the program’s ability to recruit a diverse pool of global instructors ensured that training was not constrained by local availability of expertise, maximizing the number of instructors available on short timeline, and enriching the content of the educational sessions.

Based on the implementation experience, the planning team also identified many opportunities for improvement and several steps to avoid for future programs. For example, there was an observed positive association between MOH involvement and session attendance. The first session which included an introduction by a representative from the MOH had 113 attendees. Later sessions, when MOH representatives were not available to participate, had lower attendance. Ideally the MOH would have been able to participate in all 16 of the learning sessions. Related to this is the challenge of recruiting HCW participants in a setting of war, especially with the constrained communications channels common in early phases of an active conflict. More information about best practices for recruiting participants is needed. Another opportunity for improvement was the observed very limited engagement and interaction from the audience during each session. This was felt to be related to the use of the webinar format on the Zoom platform in contrast with the meeting format which allows participants to share their name, video and audio and use their microphone. The invisible and anonymous nature of the participant experience may have limited a sense of community among the HCWs and thus restricted a willingness to communicate in each session. Was engagement by participants reduced due to the use of the webinar format versus the video conference meeting format or for other reasons? Was there a reduction in the number of live participants because of other pressing needs for the attention of HCWs during the early days of the conflict or because they were able to watch the recordings asynchronously at their convenience? Further exploration of these questions and of the true security risks associated with using a videoconference meeting format in which all participants could be seen weighed with benefits of improving engagement is needed.

## 3 The Sudan emergency ECHO

With the onset of hostilities in Sudan in April 2023, there was a recognized need for rapid emergency medicine and trauma care education among healthcare workers (HCWs). Unlike the ACTT program in Ukraine, which was initiated without a pre-existing remote digital education community of practice, Sudan already had a robust remote healthcare education and primary care support program. This HCW learning network, which also included medical and nursing students, was developed during the COVID-19 pandemic in partnership with Project ECHO at The University of New Mexico (UNNM) and had been operational across multiple states in Sudan since 2020. The program, housed within the Sustainable Development Response Organization (SUDRO)—a non-profit focused on achieving sustainable communities through partnership, knowledge exchange, and capacity building—has engaged in a broad array of community-based health strengthening activities.

As of April 2023, the Community Medical Response Team (CMRT) group, integral to this program, included of over 5,000 HCWs linked through the Telegram messenger app. This platform was chosen for its ability to engage a large number of participants with simple smartphones, requiring very low bandwidth. It also facilitates a blend of SMS and document sharing, along with the capability to launch webinars directly within the app. This setup sparked a demand for emergency care education, particularly among primary care providers and physicians-in-training.

SUDRO led a collaborative effort with Project ECHO and the WHO Eastern Mediterranean Regional Office (EMRO) to deliver trauma care and public health emergency response training to the Telegram CMRT group members. This program drew on experiences from the Ukraine ACTT initiative, intending to use the BEC trauma materials as the foundational structure for the course. However, the Sudan Emergency ECHO program also diverged in several ways from its Ukrainian counterpart. Capitalizing on the existing CMRT network, the program was able to, very rapidly, launch within 5 days. The curriculum initially focused on BEC content but was quickly adapted based on continuous assessment of field reports from HCWs, reflecting emerging priority needs. This adaptability was further enhanced by frequent reliance on Sudanese trauma care experts as instructors, who provided contextually relevant and culturally nuanced content directly in Arabic, eliminating the need for interpretation.

### 3.1 Sudan emergency ECHO observations

Between April 29th and October 25th, 2023, the Sudan Emergency ECHO, in collaboration with the WHO EMRO Case Management team, delivered 43 training sessions. These sessions included a total of 2,697 attendances with an average of 65.8 participants per session. One of the most significant strengths of the Sudan Emergency ECHO program was its delivery to a pre-established network of over 5,000 healthcare workers linked through Telegram. The advertisement of the first trauma training session in late April 2023 saw the group’s membership more than double within a week and reach over 14,000 members in the following weeks. This expansion significantly increased the number of HCWs invited to participate in programs and is believed to have positively influenced attendance of live sessions and viewing recorded materials asynchronously. Direct access to such a large group of HCWs proved highly efficient for disseminating learning session resources like PowerPoint presentations, articles, WHO guidance documents, and links to recordings. It also opened the possibility of not only rapidly and widely disseminating information, but also gathering critical information from a large group of participants related to local healthcare capacity and infectious disease surveillance to inform response efforts. Future programs should consider increasing bidirectional information sharing between program hosts and participants to further support response efforts. This direct engagement model also addressed challenges noted in the Ukraine ACTT program related to limited participant engagement and unclear outreach strategies, which had been compounded by the lack of direct guidance and involvement from the Ukraine MOH.

The use of Telegram, a low bandwidth digital communication platform, was a critical strength, allowing the program to continue without interruptions despite frequent information technology infrastructure disruptions in Sudan. The program’s model of ongoing, responsive assessment enabled swift adaptation to the changing needs of the learning community, such as shifting focus temporarily from trauma management to maternity care during a rise in home births or to cholera management following outbreaks related to water supply infrastructure disruption.

The involvement of Sudanese experts as lecturers, both locally and from the global Sudanese diaspora, enriched the program by providing direct instruction that was both clinically and culturally informed. These experts brought firsthand experience and understanding of the local context, enhancing the relevance and impact of the training provided. While partnering with local experts presented challenges related to communication infrastructure and security, these were not insurmountable and highlighted the need for a careful approach to ensure safe and reliable involvement in future programs.

Recognizing the urgent need for just-in-time emergency care expertise, a novel crowd-sourced virtual consult service was established via a Telegram messenger group specifically for healthcare workers to pose real-time clinical questions to a pool of regional trauma and emergency care experts. These experts were quickly identified and vetted by a WHO trauma and emergency medicine specialist and made accessible to healthcare workers in Sudan. However, the use of this virtual consultation group by members of the Community Medical Response Team (CMRT) was modest and much less than expected. The reasons for this limited engagement are not entirely clear, but may be due to the service being an additional component of a larger program, which possibly stretched the program team too thin to effectively foster an active dialog among HCWs. Considering this, future programs might benefit from having a dedicated leadership and coordination team specifically dedicated to managing virtual clinical consultations. Despite the underutilization of this service, we believe it represents a valuable opportunity and warrants further development and improvement in future implementations.

The Sudan Emergency ECHO program offers valuable lessons on the benefits of leveraging existing HCW learning networks and technology to deliver timely and culturally relevant emergency care education in crisis settings. It also underscores the importance of deploying emergency programs on existing virtual networks, maintaining flexibility to adapt program content, and incorporating content experts who are also familiar with the cultural and clinical context such as local experts or diaspora, when addressing the specific needs of HCWs in conflict zones. An additional benefit of delivering a emergency training on a standing digital learning network is that the resources and network of instructors can be documented and stored by program coordinators to be reactive if needed. This retention of resources and contacts provides a level of sustainability which was is not present in a program that has limited or no ongoing digital learning activities in the affected country.

In 33 of the 43 sessions a two item poll was presented to attendees through the Telegram app during the program. For question one, “How confident are you in applying information from this session?,” among respondents across all sessions (N = 1,104) 86.32% of respondents described feeling “extremely” or “moderately” confident about applying information from the session with 6.34% reporting feeling “slightly” confident and 7.34% responded feeling “not confident.” From responses to poll item number 2 (N = 986) “Tell us what you think about the session” 58.6% of respondents reported learning something new that they have either applied or are eager to apply in their work. Table 5 describes a summary of responses to the polls. During analysis of individual session data, the authors found 6 duplicated entries as well as a blank entry for 2 question responses. The responses for these questions from the corresponding individual session were removed from the data set prior to final analysis.

**Table 5:** Responses among Sudan emergency ECHO participants rating how confident they are in applying information from this session (*N* = 1,104) and to describe their thoughts of the session (*N* = 986).

How confident do you feel about applying information from this session?		Tell us what you think about the session?	
Extremely confident	59.96%	I learned something new that I have applied in my field of work	30.07%
Moderately confident	26.36%	I learned something new that I am eager to apply in my field of work	27.53%
Slightly confident	6.34%	I was reminded by things that I already knew that are related to my field of work	11.09%
Not confident yet	7.34%	Unfortunately, I have not learned anything new	2.29%
		Unfortunately, I did not attend this session	28.01%

Due to the rapid pace of program development and the planning team’s focus on curriculum, a formal evaluation tool wasn’t available in the initial program sessions. Starting on July 27th, 2023, demographic data were gathered from attendees in addition to poll responses. Moving forward, it’s imperative to plan and implement data collection methods for evaluating programs prior to the launch of similar digital learning initiatives.

## 4 Discussion

The Ukraine ACTT program and the Sudan Emergency ECHO program present similarities and differences in their approaches to deploying remote emergency care training programs. Four critical elements were common to both initiatives: 1. A shortage of functional healthcare facilities capable of treating patients, 2. An increase in emergency cases, including trauma, 3. A knowledge gap in trauma care and public health emergency response among local healthcare workers (HCWs), and 4. A reliable telecommunications infrastructure for reaching and communicating with HCWs. The absence of any of these elements would compromise the program’s benefits and the rationale for pursuing similar initiatives. For instance, following the outbreak of hostilities in Gaza, the collapse of the healthcare system resulted in a lack of essential resources like food, water, and electricity. In such extreme conditions, prioritizing remote health systems strengthening activities becomes less critical compared to meeting basic needs (13). However, if the needs of HCWs align with the critical elements mentioned above during a conflict, a remote virtual education program can be strategically deployed as part of a broader effort to rapidly enhance healthcare emergency response capabilities in active conflict zones.

Each of the programs was rapidly developed in response to urgent and ongoing crises. The coordinating teams were primarily focused on providing essential educational resources to healthcare workers in active conflict zones. Consequently, attention was largely directed toward preparing the programs for effective delivery rather than prioritizing comprehensive data collection efforts. However, in each program, attempts were made to gather data on participant experiences. The Ukraine ACTT program benefitted from participant evaluations conducted after every session, whereas the Sudan Emergency ECHO program had more limited evaluations, with expanded data collection beginning several months after the program’s launch. In the future, digital learning initiatives tailored for acute humanitarian emergencies, such as conflict zones, should incorporate data collection tools from the outset and comprehensive evaluation at the completion of the training to more thoroughly evaluate the efficacy and impact of such programs. Importantly, prioritizing security considerations for participants should be considered and reevaluated when conceiving and delivering such programs.

The lessons learned from both the Ukraine ACTT and Sudan Emergency ECHO programs can serve as a foundation for designing future initiatives. Feedback from participants and coordinators, though limited, indicates that these programs were valued, suggesting a net positive impact and potential for replication and improvement under similar circumstances.

The aim of this paper is to disseminate two case examples of ways to use technology to rapidly deploy training and education for healthcare workers in conflict areas and in complex emergencies. In crises where local healthcare workers have limited training or experience in emergency care, digital health care education can be rapidly delivered independent of, or to augment in-person response and training efforts with minimal risk to additional healthcare resources. While recognizing that the quality of in person training may not be matched with remote training, these case examples provide important examples of the strengths and challenges associated with the development and delivery just-in-time learning programs in active armed conflict settings and suggest similar potential utility in a variety of humanitarian emergency contexts.

## 5 Conclusion

The 76th World Health Assembly calls for a range of actions by member states to improve emergency care including the promotion of “inclusive and accessible approaches to safeguard effective emergency, critical and operative care in disasters, fragile settings and conflict-affected areas” (1). Effective response to humanitarian crises requires robust on-the-ground action and precise assessments to prioritize responses. Given that local HCWs often perform critical clinical work during such emergencies and may lack prior training in emergency medical care, learning and capacity strengthening are essential to ensure a strong and effective response. While in-person training offers numerous benefits and remains indispensable for certain aspects of emergency care, remote training can significantly enhance and expedite capacity building through education, training, and ongoing bidirectional information sharing.

The Ukraine ACTT and Sudan Emergency ECHO have demonstrated success in rapidly deploying remote just-in-time capacity strengthening strategies and highlighted areas for improvement. Future efforts should focus on improved monitoring and evaluation strategies to better assess the strengths and weaknesses of these approaches. A thorough evaluation of program effectiveness is crucial. By embracing and enhancing remote learning and capacity strengthening, along with traditional response models, the international health community, leveraging the expertise of in-country and global diaspora professionals and additional global experts as appropriate, can make significant strides toward achieving more coherent, inclusive, and accessible approaches to ensure effective emergency, critical, and operative care in disasters, fragile settings, and conflict-affected areas. These initiatives can help minimize loss of life and reduce suffering and answer the call of the 76th World Health Assembly.

## Data Availability

The raw data supporting the conclusions of this article will be made available by the authors, without undue reservation.
